# Waiting with Beckett in the Anthropocene

**DOI:** 10.3366/jobs.2024.0415

**Published:** 2024-04

**Authors:** Laura Salisbury

Just a month before the end of Samuel Beckett’s long life, the Berlin Wall finally came down. Now living in a care home, Beckett watched the television footage. But instead of celebrating, we hear that he was agitated. ‘Ça va trop vite’ (qtd in Cronin, 1996, 591), he kept saying. Instead of imagining that the effects of political crisis would produce historical change and progress, Beckett, it seems, glimpsed catastrophe. Placing to one side whether Beckett was right on the speed of change for those who had been waiting twenty-eight years for the Wall to fall, the vignette resonates because it repeats a set of aesthetic and ethical preoccupations in Beckett’s work: the long-held suspicion that forms of quick action that are represented as progress, achievement, or gain, risk mortgaging the embodied particularity and suffering of past and present to an idea of a changed future. As we continue to read and stage Beckett beyond his own time, amidst ecological crisis and the urgent call to action, what is to be made of Beckett’s slowness and emphasis on waiting? How might we read the attachment to the ‘nothing to be done’ (Beckett, 1990, 11)?

The concept of the Anthropocene, first coined in 2000 by Paul J. Crutzen and Eugene F. Stoermer, proposes that humans should now be understood not simply as agents within historical time, but producers of change at a geological, planetary level. The Anthropocene’s beginnings have since been variously aligned with key points within the developmental timelines of modernity: the colonisation of the Americas (Moore, 2015); the Industrial Revolution (Lewis and Maslin, 2015); the emergence of intensive agriculture, especially that undergirded by enslaved labour and plantations (Haraway, 2016); and the plutonium fallout from nuclear weapons use and testing detectable across the planet from around 1952 (Waters et al., 2015). Framing the refusal to face up to the destructive effects of modernity’s temporalities of growth, accumulation, and progress as a failure of care and action at an individual and global scale, Amitav Ghosh implicitly invokes the crisis urgency of a ticking clock: ‘every year that passes without a drastic reduction of global emissions makes catastrophe more certain’ (Gosh, 2016, 160). Even Rob Nixon’s influential argument that much of the attrition of the climate emergency does not display itself as a spectacular event, following an easily recognisable dramaturgic or historiographic form, is underpinned by the sense that something must be done: writers must ‘devise arresting stories, images, and symbols adequate to the pervasive and allusive violence of delayed effects’ (Nixon, 2011, 3). There is, quite simply, no time to wait.

Despite the clear resonance between Nixon’s call to attend to ‘long dyings’ and Beckett’s work, this essay argues that Beckett’s writing provides few resources for the time-critical project of ‘turning the long emergencies of slow violence into stories dramatic enough to rouse public sentiment and warrant political intervention’ (Nixon, 2011, 19–20). True, Beckett’s rhythms resist the tempos of progress that have powered the engine of global history and are seen as proper to the specific category of the human that emerged in the West alongside the Enlightenment and what Jason Moore calls the ‘Capitalocene’ (2015); but they also resist the timelines of crisis invoked by efforts to transcend them. Perhaps there is something in staying with this resistance, though, with its repetitions, boredoms, and refusal of gain. Leo Bersani’s intriguingly minimal description of an ‘ecological ethics’ as ‘one in which the subject, having willed its own lessness, can live less invasively in the world’ (Bersani, 2010, 62), implies that what Bersani and Ulysse Dutoit named Beckett’s ‘art of impoverishment’ (1993) has something to say to a planet struggling with legacies of invasiveness, extractivism, accumulation, and relations between and within human and more-than-human worlds where sustenance, growth, and expansion cannot be taken for granted. Reading the idea of the willed lessness in Beckett’s later work through the return to what becomes vulnerable to the ‘trop vite’ – the specificities of suffering bodies over time that keep on pulling the human, as a ‘subject of knowledge’ (Bersani, 2010, 62), back to qualities of matter that refuse its projects – I will suggest that Beckett’s temporality offers neither a vision of hope nor a cautionary apocalyptic tale that would turn back the clock. Instead, Beckett’s late fictions offer minimal imaginative glimpses of waiting bound to care that interrupt and suspend the spatialised timeline of progress but attend instead to enduring in the meanwhile.

## The Sense of the End Times

As a response to the idea of climate change as both a problem of time and of representation, the Anthropocene is shot through with the sense of an ending. Crutzen and Stoermer insist that action to protect ‘ecosystems against human induced stresses’ in the face of a projected denouement and end that will be experienced on a planetary level, is ‘one of the great future tasks of mankind’ (Crutzen and Stoermer, 2000, 18). In the narrative of the Anthropocene, a tipping point has already been passed. The marks on the stones and bones of human and more-than-human worlds are both indelible and increasing in scale and frequency. What has been called the ‘Great Acceleration’ of the 1950s – the period when the slow refusals of Beckett’s mature vision crystallise – was profoundly marked by new accumulations and distributions: the increasing material conversions of fossil fuels; the dissemination of carbon particles worldwide; new geochemical compounds and pesticide residues; the doubling of soil nitrogen and phosphorus due to artificial fertilisers; and dispersals of materials like aluminium, concrete, plastics, and synthetic fibres across the globe (Yusoff, 2019, 30). Without any slowdown in these forms of world-making and in the face of the imminence of existential threat, there come to be two intransigently linear possibilities for ending within the plot of the Anthropocene: either lasting reform of humanity’s ways of going on through remedial and anticipatory action (Anderson, 2010), or extinction, whether quick or slow.

As Frank Kermode has argued in *The Sense of an Ending*, narratives, whether literary or otherwise, work to lift people out of the middle of things, allowing them to inhabit the beginning and to project themselves beyond the end in a way that offers structure to the whole (Kermode, 2000, 8). For Kermode, time as *chronos* – ‘passing time’ or an empty ‘waiting time’ – is bisected by *kairos*: ‘a point in time filled with significance, charged with meaning from its relation to the end’ (47). For Kermode, fictive ends frequently borrow from eschatological frames that bring ongoing time to an end through an atemporal perspective. The Anthropocene imaginary also repeats Christian narratives that focus on judgment and then salvation or damnation to invoke a sense of living through times where the end is nigh (Rothe, 2019). But alongside this sense of Messianic time with which the human must urgently align itself, the Anthropocene also uses modernity’s turn from eschatology to the time of prognosis, intervention, and crisis in which human action is understood as creating an open future by seizing and turning history (Koselleck, 2004). Apocalyptic disasters are imagined that ‘we’ are somehow still present to witness; ‘we’ are thus called on to produce the anticipatory action to fashion other kinds of ends.

As numerous critics have argued, the Anthropocene suggests an apocalyptic imaginary in which those in the West, the Global North, and who have benefitted from settler colonialism and industrial modernity, imaginatively project themselves beyond an end in order to mourn the loss of their way of life, without considering how the ‘end of the world’ has already been produced and lived: by Indigenous people, many in the Global South, and racialised peoples whose lives and environments have been appropriated, harnessed, and destroyed for the benefit of others (Wynter, 2015; Yusoff, 2019). Grace Dillon has asserted that the ‘Native Apocalypse, if contemplated seriously, has already taken place’ (2012, 8), while in his critique of the overreliance on models of linear time to describe the climate emergency, Kyle Powys Whyte argues that although ‘Linear measures of time have the capacity to generate a sense of imperilment and urgency’ (Whyte, 2021, 45), the ticking clock misses other figurations of time beyond the interests and tempos of those humans who have historically framed themselves as able to produce, mark, or outlast ‘the end’. Claire Colebrook follows this line, noting that ‘certain people can meet their end, but those extinctions are not the end of *the world*’ (Colebrook, 2019, 279). All that falls or is pushed outside of a historically and culturally specific idea of the sovereign human subject can end before the end of the world is framed as operating at a planetary level: as universal, revelatory, and *kairotic*. And if the idea of the planetary can only be invoked when particular configurations of the human are threatened, there is little sense that the productionist, progressive, anticipatory logics and tempos that have initiated and sustained the Anthropocene will be decentred by writing ends where only a certain version of ‘man’ is left as a witness (Allen-Paisant, 2021, 48).

Writing in the early 1960s, in the cold shadow of seemingly imminent nuclear apocalypse, Kermode noted that the idea of fiction and poetry as easily ‘time-redeeming’ (Kermode, 2000, 52) had stalled. In the face of the possibility of annihilation that could simply end human time, and without a religious frame giving eschatological meaning to Apocalypse, the idea of a revelation witnessed by God, the saved, or the damned was significantly interrupted. ‘[W]e no longer live in a world with an historical *tick* which will certainly be consummated by a definitive *tock*’ (64); instead, there was and is the threat of a cataclysm that will leave no one to shape and narrate it. Kermode does not read Beckett’s work here, but his endings are clearly not ‘time-redeeming’, working as revelations, whether transcendent or destructive. There are witnesses left in Beckett’s scenes, but something resists representation. As Bersani and Dutoit argue, ‘Endings are at once certain (they are inscribed in beginnings) and indeterminable’ (Bersani and Dutoit, 1993, 46). An ending does not and will not come to redeem time’s empty passage; endings multiply and are multiply outlived: ‘The end is in the beginning and yet you go on’ (Beckett, 1990, 126), as Hamm says in *Endgame*. The answer to ‘what’s happening?’ is that something is always ongoing; ‘something is taking its course’ (Beckett, 1990, 98). Or, as Beckett writes in ‘Le monde et le pantalon’, ‘Tout cesse, sans cesse’ (1984, 128).

*Endgame*’s ‘slow work’ of waiting in a world considerably more imperfect than a pair of trousers, botched in the making, can be figured as a form of living on in the wake of disaster. There is no ‘beyond the hills’ where ‘Perhaps it’s still green. Eh? Flora! Pomona! Ceres!’ (see Garrard, 2011); instead, all comes to resemble the vision of the past ‘madman’ Hamm invokes, who, when faced with a sight of natural plenty and growth harnessed to support the human – ‘All that rising corn! […] The sails of the herring fleet!’ – can only avert his eyes, appalled: ‘All he had seen was ashes’. In this ‘Old endgame lost of old’, life can only ever be ‘the life to come’ (Beckett, 1990, 97, 103, 111, 113, 132, 116). The slowly accumulating mound in which Winnie in *Happy Days* is encased will similarly never bring the mercy of quick extinction or cure, only ongoing slow violence following a catastrophe that has already taken place, beyond our purview. ‘Man adapts’, or, more specifically, woman adapts, only (Beckett, 1990, 153). In *Malone Dies*, we are resolutely not at the beginning, for the eponymous narrator notes that at times ‘the earth seems uninhabited […] you begin to fancy yourself the last of human kind’ (Beckett, 1994, 254). But Malone, whose narratives of ending persist ceaselessly, shows that we can’t expect an imminent end either. Characters and readers are instead cast into a meanwhile: enduring in the wake of a former life while waiting in the shadow of a life to come that is also a loss to come.^2^ But although the markers of historical or linear time have been withdrawn and even circadian and seasonal rhythms have stuttered, time still passes, marked within the to and fro between characters, or a sense of exchange with a witnessing audience or reader. Time passes while waiting, even though, as *Godot* reminds us, ‘It would have passed in any case’ (Beckett, 1990, 46).

When particular figurations of the end dominate, there is a narrowing of attention on to an imagined future. Anna Lowenhaupt Tsing has argued that ideologies of progress, including the notion of movement towards a desired or feared end, reshape the material world by seizing and making use of time; and they are detrimental to cultivating diverse temporal ‘arts of attention’ (Tsing, 2015, 21). As Claire Colebrook articulates it, the Anthropocene has

intensified not only the *desire* for a linear future (a future that would follow from how we imagine life *ought* to be, a future that would be the outcome of a free and considered decision rather than whatever may come to pass), but also a prediction that there must actually be the possibility of a future that would be in accord with our intentions. (Colebrook, 2018, 109)

But climate science and systems theory have shown that linear time is not so biddable. It is impossible to predict causes and effects with certainty and to know that a specific action in the present will produce a desired outcome. Instead, there are multiple futures that can open from the ‘volatility of the present’ (Colebrook, 2018, 109), or, as Tsing proposes, new paradigms beyond the idea of a singular end where time becomes complexly interconnected and multi-directional.

Instead of moving with the tempos of emergency or crisis action orientated towards projected ends, it might then be important to be prepared to wait and to attend carefully to multiple lines of time. These might include the timeline of progress and human history, but also other lines that are neither predicated upon, nor necessarily include, ‘our’ survival (Colebrook, 2018, 118). Beckett’s work is certainly interested in what remains – in how bodies, things, and even environments linger as residues of what once had use value – but they are not cautionary tales of environmental degradation; they are, instead, bound to representing a scene in which the autonomy and agency of the human, and the relations between the human and more-than-human, are not what they were. In this grey vision it is paradoxically clear that both human and the more-than-human worlds operate in ways that are, through and over time, running otherwise to the force and capacity of the human as a synecdoche for reason, agency, sovereignty, and intention. Instead of the human becoming an agent within deep time, other timelines, which include but are not limited to the geological, interrupt the time of progress and action.

Colebrook suggests that one way to decentre the immanent temporal logic of the Anthropocene would be to attend to the fragmentation of time into multiple lines. Here, she builds on Gilles Deleuze and Félix Guattari’s monist idea of the plane of consistency, described in *A Thousand Plateaus* as the ground of all becoming. This plane is a site of immanent force which generates and sustains the multiplicity of forms through networks of particles, links, affects, and relations (Deleuze and Guattari, 1992, 266). In this context, time is neither rectilinear nor homogeneous; it is formed through a multiplicity of speeds and intensities that intersect, overlap, and are folded into one another. But whereas Deleuze and Guattari’s temporal multiplicity is generally understood as an open horizon of becoming for ‘us’, Colebrook suggests dwelling with how intensity breaks apart singular lines of time, forcing an awareness of what is deadened within trajectories and tempos of development, including that which has been figured as more than or less than human:

Rather than a humanity that embraces a nonlinear time and finds hope in the most open of futures, there would be futures that are not our own and that unfold from all the lines of the past that have been deadened, buried and held in abeyance by the celebration of an extensive rather than intensive multiplicity. As extensive, humanity can always add variants, differences and becomings to its open and creative time, and always – by doing so – hold on to a future. Thought intensively, every line of time has a future that destroys the coherence and unity of the present; the lines of time – in their singularity – destroy both any single line of time, *and* any notion of an open future *for us*. (Colebrook, 2018, 118)

In what follows, I will pursue the suggestion of a kinship between the way matter and relations, thought intensively, produce forms that challenge the dominance of the world-forming powers of the *anthropos*, and the way time, thought intensively, calls attention to that which cannot be subordinated to the lines that underwrite a future for the human subject as it has articulated itself in modernity. The suggestion will be that Beckett’s work, analysed in relation to these intensities, opens up modes of waiting and forms of temporal attention that produce a stutter – sometimes an interruption, sometimes a thickening – in rectilinear time. Instead of a ticking clock of time running out in which ‘taken-for-granted strategies get employed to protect the taken-for-granted state of affairs from disruption’ (Whyte, 2021, 49) and only a future for some is preserved, Beckett’s particular ‘art of attention’ forces a return to the time of attending, of waiting, and a scene of matter and of mattering, alongside the questions of care that inhere with them.

## Anachromism

In her reading of the figure of Greta Thunberg as an articulation of the temporal demands of the Anthropocene, Lisa Baraitser (2020) alights on the version of time that freights a call to action. Thunberg asserts that we are simply out of time; we need to make a choice if we are to have a future:

They keep saying that climate change is an existential threat and the most important issue of all. And yet they just carry on like before. If the emissions have to stop, then we must stop the emissions. To me that is black or white. There are no grey areas when it comes to survival. Either we go on as a civilization or we don’t. (Thunberg, 2019, 6)

But Baraitser brings Thunberg’s insistence on the value of black and white thinking – the refusal of impasse, suspension, and waiting – into dialogue with precisely the grey she disavows: a ‘grey time’ where there is neither simply action nor its opposite, but a call to keep paying attention to the intensity of time that resists the black and white of a crisis (Baraitser, 2020, 513). This idea of grey time, which Baraitser draws from my thinking about Beckett’s aesthetics, suspends the ticking clock and the crisis temporalities of the Anthropocene, but it does so, at least potentially, not as a defensive escape from the reality of destruction and damage, but as a way of attending to it even in the face of the foreclosure of the future.

In 1961, Beckett described his work as inhabiting a grey zone:

If life and death did not both present themselves to us, there would be no inscrutability. If there were only darkness, all would be clear. It is because there is not only darkness but also light that our situation becomes inexplicable […] The question would also be removed if we believed in the contrary – total salvation. But where we have both light and dark we have also the inexplicable. The key word in my plays is ‘perhaps’ (qtd in Driver, 1961, 23).

As I have argued elsewhere (Salisbury, 2023b), Beckett’s environment admits neither the certainties of black (the absence of transmitted or reflected light), nor white (the transmission or reflection of light at all visible wavelengths). Instead, things are ashen: ‘GRREY! […] Light black. From pole to pole’ (Beckett, 1990, 107), as Clov insists. In *Happy Days*, there is no real hope of an ‘eternal dark. [*Pause*.] Black night without end’ bringing the mercy of extinction (Beckett, 1990, 166); nor does any brilliant, enduring white that Beckett’s minimal cylinder experiments like *Ping* momentarily offer last. White becomes obscured: ‘white planes shining white bare white body […]. Traces blurs signs no meaning light grey almost white’ (Beckett, 1995, 193).

As Daniela Caselli argues (2005), the figure of the child in Beckett’s work, like the small boy Clov spies through his telescope emerging from the ‘light black’ (Beckett, 1990, 130), is not representative of the putative clarity and spontaneity of the child’s perspective – the black and white simplicity on which the rhetorical force of Thunberg’s intervention as a child leans. Beckett’s small boy is not a figure of the future offering either salvation or extinction; he just emerges momentarily from the grey, only to return to it, giving shape to a time that shudders through this.^1^ In this grey time, there is little sense of a progressive movement towards what we might think of the enlightened promises of modernity; but neither is there a possibility of the blank blackness of obliteration or extinction bound into modernity’s timelines of industry, extraction, and war. Instead, grey time materialises a waiting without either cure on the horizon or death as a release.

Held in an impossible time of chronic, ceaseless dying, Malone indeed describes how the space in which he is immured is bathed in a ‘kind of leaden light that makes no shadow, so it is hard to say which direction it comes, for it seems to come from all directions at once, and with equal force […]. I even sometimes have the feeling that I emit grey’ (Beckett, 1994, 221). Malone notes ‘there really is no colour in this place, except in so far as this kind of grey incandescence may be called a colour’ (221), and, of course, grey is not truly a colour at all; it is, rather, a shade. Grey is achromatic: composed of black and white in various shades of intensity rather than hues. When grey, which is neither simply black nor white, is thought in relation to time, it might be described using a neologism: we might say it is ‘anachromistic’. In Beckett’s grey we can trace a temporality that has a kinship with anachronism – a chronological inconsistency that is ‘against time’ as it is usually arranged. To speak of anachromism suggests Beckett’s return to a strictly limited palette and defection from affective ‘colour’ (Salisbury, 2023b, 53–4); but, as with anachronism, grey time also produces a slub in the fabric of time, interrupting or suspending its rectilinear continuity. Anachromism marks time’s intensity and variegations within moments that are suspended, rather than, as is more usual, describing its flow, direction, or progression. Anachromism pays attention to Beckett’s weaving in of blank, uncertain, colourless ‘colour’ and affect into what is felt of time.

Beckett renders his anachromistic temporality of grey-on-grey palpable across his texts and their embodied reception by exploring stilling, though never still or completely indifferent, shades of difference in which black and white are forced to remain in contact with one another: here intensifying, there dissipating. As the narrator of *The Unnamable* puts it:

Whether all grow black, or all grow bright, or all remain grey, it is grey we need, to begin with, because of what it is, and of what it can do, made of bright and black, able to shed the former, or the latter, and be the latter or the former alone. (Beckett, 1994, 303)

Rather than chromatic difference or the difference in kind that enables a sense of contrast, dialectical progress, or the passage of time, grey time accumulates in differing shades of intensity. Malone’s room is indeed described as ‘the same grey as heretofore, literally sparkling at times, then growing murky and dim, thickening is perhaps the word, until all things are blotted out’ (Beckett, 1994, 223). Time gathers in an ‘impossible heap’ (Beckett, 1990, 93). As contrasting differences that enable forms of dialectical temporal progression become difficult to detect, Beckett materialises grey-on-grey instances of intensity that emerge while waiting in a suspended yet ongoing time.

Beckett’s *Lessness* contains his most insistent return to grey. This is partially due to its compositional structure, which is dependent on the randomised sequencing and arrangement of pre-written sentences that in the published version is run through twice, though it could repeat endlessly (Cohn, 2005, 305). The sense of a snapshot of a potentially endless sequence is intensified by the text’s repeated ashen imagery. The space of ‘refuge’ that might have offered the peace of what is ‘Blacked out’ has ‘fallen open’, while the possibility of ‘all light white calm [has] all gone from mind’ (Beckett, 1995, 199). The black is shifting – it is ‘slow black’, moving towards grey: ‘Grey sky no cloud no sound no stir ash grey sand. Little body same grey as the earth sky ruins only upright. Ash grey all sides earth sky as one all sides endlessness’ (197–8). On each page and in multiple configurations within sentences laid down like strata, the text tells us that the grey has a suspended temporality: ‘grey air timeless’ (197–201). But the material text as produced, published, and read by material bodies invokes a time that inches forwards rather than repeats endlessly. There is an evocation of the intensity of articulations of lessness in the shadow of endlessness: an anachromistic grey of modulating intensities produced by the passage of time within strict formal constraints in terms of the textual resources at its disposal. This willing of lessness, as Bersani might have it, suggests both a defection from artistic practices based on endless innovation and the excavation of new resources. In that sense, there is an ‘ecological’ and ‘ethical’ resistance to expansion and to what might be thought of as the colonisation of new artistic territory. But rather than such lessness becoming a strategy leading to straightforward aesthetic or ethical gains, working within strictly defined limits where the only resource proliferating is the unspooling of waiting time holds attention to the ongoing suffering of particular bodies enduring within the move towards lessness. In this mode of artistic extraction, each germ of value within lessness is therefore understood as both a gain and a loss.

## Death Driven

‘For to end yet again’ (Beckett, 1995, 243). This *Fizzle* from 1976 feels familiar, though there are always differences that glimmer and shade amidst the seeming indifference. In this anachromistic landscape of shades, passing on or passing over seems foregone, even though different configurations of intensity enable things to shudder into view: ‘Grey cloudless sky verge upon verge grey timeless air of those nor for God nor his enemies’ (Beckett, 1995, 244). Here, Beckett holds us in the grey time of ‘perhaps’, suspended between salvation and damnation (using his terms), with a figure, a body, placed once again in the ruins of a crumbling refuge that can no longer separate interiority from the outside into which all seems to be falling. There are flashes, revenants of past texts. The figure, ‘the expelled’, is encased in a container of sand, recalling *Endgame’s* Nagg and Nell, while the ‘timeless air’ and the ‘ruins of the refuge’ return us most clearly to the environment of *Lessness*. The figure here is like a monolith – ‘the arms still cleave to the trunk’ (Beckett, 1995, 243) – and there is no face or neck to be seen, only glimmers of eyes, occasional strikingly clear particulars in the box of a skull. The exact qualities of the scene we are witnessing never become fully distinct. Is the head attached to the body, or has it toppled like Shelley’s Ozymandias? The scene never clarifies enough for the eye to differentiate fully one grey shape from another. That Beckett may have been thinking of Ozymandias, as Mark Byron has noted (2020, 22), is perhaps confirmed as we hear of the ‘Sand pale as dust ah but dust indeed deep to engulf the haughtiest monuments which too it once was here and there’ (Beckett, 1995, 243). Maybe this is an ironic statement on Beckett’s own increasingly monumental body of work that is nevertheless decrepit and despairing – perhaps even derivative. For here the disintegration of the work as a metonym for a belief in human agency – ‘Look on my Works, ye Mighty, and despair!’ – is where we begin, though the despair is rather more indifferent than the one Shelley imagines.

The refuge here is, roughly, a cylinder, reminiscent of the almost science-fictional pieces of the 1960s in which grey bodies endure in the grey light of a half-life. But now, as in *Lessness*, the walls of the ‘refuge’ are becoming ‘ruins’ (Beckett, 1995, 244), losing their integrity and falling outwards. So, there is change: things are not yet still or extinct: ‘First change of all in the end a fragment comes away and falls’ (245). Within this particular end, however, something different emerges. Two white dwarfs are imagined carrying a litter – a stretcher. Both dwarfs and cargo are barely distinguishable from the litter of stones and sand in the bone-white/grey of the scene. But these dwarfs and their litter – which of course can also represent non-human animal offspring – are something new in Beckett’s universe. Perhaps they represent a primordial or mythological Teutonic or Scandinavian past; but, as Ackerley and Gontarski note (2004, 204), a white dwarf is what Earth’s sun will become when it has exhausted all its nuclear fuel. Dense and dim, white dwarfs are stellar corpses waiting for heat death: the entropic future of our solar system. In this *Fizzle*, these dwarfs and their litter offer at least the potential to carry away the grey body – whether for the purposes of salvation or extermination – but they also seem to represent a future of loss, even deformation. In a difficult moment for readings of disability in Beckett’s work, the text’s anatomisation of their ‘stunted legs and trunks monstrous arms stunted faces’ (Beckett, 1995, 244) matches the entropic decline of a solar system with what might be read as an invocation of species damage, even phylogenetic degeneration.

Adam Piette (2016) has suggested that Beckett’s cylinder pieces of the 1960s can be read as an exploration of ‘deep time’ in which bodies endure alongside nuclear radiation; and perhaps the half-lives imagined here also represent legacies of genetic damage and waiting beyond remembering. If this is indeed a scene invoking the deep time violence of nuclear radiation in the Anthropocene that sutures a distant future to the time of its composition, there is value in teasing out other science fictional intertexts, including H. G. Wells’s 1895 *The Time Machine*. Wells’s Morlocks are of short stature, tending and feeding from the other evolutionary off-shoot of modern humans, the agency-less white Eloi, in a nightmare vision of the extractive processes of modern labour, subsistence, and reproduction. The Time Traveller first spies a Morlock, which resembles ‘some greyish animal’, as he witnesses an ‘ape-like creature […] carrying some dark body’ away (Wells, 1935, 50, 51). *The Time Machine* also ends with the Time Traveller moving forwards so far in time that the end of all life is glimpsed, and being falls back into unbeing. Mammals ‘degenerate’ into phylogenetically earlier giant crabs, London becomes a ‘salt Dead Sea, the stony beach’, while in the final glimpse of disorder and heat death, even the tides and planetary orbits have ceased: ‘the earth had come to rest with one face to the sun’ (95, 93). As in Beckett’s *Endgame*, ‘There’s no more tide’ (Beckett, 1990, 122): all cyclical returns have ceased in the stillness of entropic disorganisation.

Phil Baker has argued that Beckett’s work stages a movement towards entropic unbeing manifested in ‘two terminal extremes each with their own satisfactions’: ‘indifference or stillness, rubbish and stone’ (Baker, 1997, 137). But in ‘For to end yet again’, the ontological distinction between disorganisation and petrifaction, sand and stone, disintegrates. For sand can become stone; stone sand. They have different kinds of intensity – density and weight, distribution and disintegration – amidst the indifference, but nevertheless suggest a kinship of matter and potential convertibility. Indeed, as in Beckett’s poem from 1948, ‘je suis ce cours de sable qui glisse’, there is an ongoing movement between stone and sand: ‘my way is in the sand flowing / between the shingle and the dune’ (Beckett, 2012, 118). Even when ‘Universal stone’ is glimpsed in the late *Ill Seen Ill Said*, it is persistently overwritten – disintegrated, one might say – by ‘One moment more’ (1992, 91, 97).

Finally, the expelled, like a monolith although a ‘Little body’ (Beckett, 1995, 245), topples; he falls back, eyes skyward, and the dwarfs become like marble statues, with the litter abandoned. The text then asks: ‘Sepulchral skull is this then its last state all set for always litter and dwarfs ruins and little body grey cloudless sky’? And the answer given is ‘no’. If the text dreams of ‘a way in space with neither here nor there where all the footsteps ever fell can never fare nearer to anywhere nor from anywhere further away’ (246) – a dream perhaps reminiscent of Clov’s dream of order under the ‘last dust’, which would represent absolute entropic disorganisation (Beckett, 1990, 120) – then, no, there is neither extinction nor salvation to be achieved, just an endless end that undermines the possibility of conventional human action. What remains is a more distributed agency and a timeline of waiting in which there is always the possibility of ‘yet another end beneath a cloudless sky’ (Beckett, 1995, 255).

‘For to end yet again’ begins by imagining a ‘skull alone in a dark place’, a ‘Place of remains where once used to gleam in the dark on and off used to glimmer a remain. Remains of the days of the light of day’ (Beckett, 1995, 243). There is an echo here of a Freudian term *Tagesrest* – the ‘remains of the day’ – which, in the *Interpretation of Dreams*, are the day’s memory traces that glimmer in the skull and are raw material for the unconscious wishes that drive dream work. For Freud, dreams are articulations of wish-fulfilment, but in 1920 Freud revised and complicated his idea of the universal dominance of the pleasure principle with the addition of the idea of the death drive, which he later described as one of the unanalysable ‘bedrocks’ [*Felsens*] of psychic life (Freud, 1955b, 252; Baraitser, 2020, 507). In *Beyond the Pleasure Principle*, Freud finds in the compulsion to repeat a fundamental ‘*urge inherent in organic life to restore an earlier state of things*’ (Freud, 1955a, 35) – a desire to return to ‘the quiescence of the inorganic world’ (62). As Baker noted, Beckett told Gottfried Büttner in 1967 of an affection for stones and even a desire to build nests for them, placing them in trees to protect them from the sea (Baker, 1997, 139). In Büttner’s paraphrase, Beckett spoke of an affinity with Freud’s idea that ‘man carried with him a kind of congenital yearning for the mineral kingdom’ (Büttner, 1984, 139). In these tantalising glimpses, we see Beckett unworking a fundamental distinction between the animacy of human life and what is imagined to be its other by placing a psychic bedrock, the ‘mineral kingdom’, within the structure of ‘man’, while suggesting that the need for care – for a nest or refuge – might extend beyond what is conventionally figured as living.

In the 1930s, Beckett encountered a version of Freud’s death drive in Otto Rank’s *The Trauma of Birth* (1924). This book can be linked to the intrauterine memories Beckett recovered during his therapy with Wilfred R. Bion (Knowlson, 1996, 177), and Beckett himself noted down this phrase from Rank: ‘Just as all anxiety goes back to anxiety at birth (dyspnoea), so every pleasure has as its final aim the reestablishment of the primal intrauterine pleasure’ (qtd in Feldman, 2006, 107). This unbinding of a simple opposition between Eros and Thanatos is of course fundamental to Freud’s account of the death drive. For the instincts of mastery and self-preservation are not simply working in opposition to death; instead, as Freud writes:

They are component instincts whose function it is to assure that the organism shall follow its own path to death, and to ward off any possible ways of returning to inorganic existence other than those which are immanent in the organism itself. […] [T]he organism wishes to die only in its own fashion. Thus these guardians of life, too, were originally the myrmidons of death. Hence arises the paradoxical situation that the living organism struggles most energetically against events (dangers, in fact) which might help it to attain life’s aim rapidly – by a kind of short-circuit. […] One group of instincts rushes forward so as to reach the final aim of life as swiftly as possible; but when a particular stage in the advance has been reached, the other group jerks back to a certain point to make a fresh start and so prolong the journey. (Freud, 1955a, 39)

In the account of the death drive that Beckett read, Rank describes how the death drive

strives again to establish nothing else but the already experienced condition before birth, and the ‘compulsion to repetition’ arises from the unquenchable character of its longing, which exhausts itself in every possibility of form. The process is biologically speaking what we call ‘life’. (Rank, 1929, 196)

So although repetition is a way of negating life, development, and change by ‘restor[ing] an earlier state of things’, it also contains elements of perseverance, preservation, and endurance that work through a tendency to shift and deviate, prolonging the journey of life in a way that exhausts itself ‘in every possibility of form’, rather than effecting a speedy dissolution.

In *Time Driven*, Adrian Johnston anatomises this bifurcated, differential quality within Freud’s metapsychological idea of drive. Johnston argues that the death drive is split because it works temporally rather than as a spatial structure; it is committed both to repetition and to deviation and development. As Johnston puts it: ‘*Trieb* is split along the lines of two irreconcilable, incompatible axes – an *axis of iteration* (source-pressure) and an *axis of alteration* (aim-object)’ (2005, 149). The desire to repeat as a way of returning to the quiescence of the world before life – ‘the mineral kingdom’ – immediately falls into the paradox of repetition that Gilles Deleuze and others have identified, and that Steven Connor (1988) has argued is at work in Beckett’s texts. Exact repetition is always doomed to failure because of the temporal element through which its status as repetition is guaranteed. As Johnston argues: ‘The axis of iteration’s demand for a pure repetition unaffected by temporal mediation can never be satisfied, since it necessarily passes through the matrix of the axis of iteration’s temporal processes’ (Johnston, 2005, 150). The commitment to iteration in the organism is a railing against time’s onward pull: away from the loss of an initial self-identical relationship of plenitude where there is no gap between subject and object, and the initial satisfaction in seeming to recover that relationship. As soon as the drive is in operation and the organism is moving through time, it will need to alter its aim-object, submitting to the substitutions of representation, and subjecting itself to the drive’s equal demand for alteration to achieve any satisfaction. This alteration emerges in the face of the impossibility of absolute repetition in time, or any return to self-identical plenitude, as the organism is exposed to the reality of experiences marked and structured by difference.

Lisa Baraitser uses Johnston’s account of the death drive’s structural commitment to iteration and alteration to develop a theory of a ‘maternal death drive’ that sustains a relationship to ‘life’ as a form of ongoing time, but remains ‘otherwise’ to the life drive and pleasure principle. Baraitser finds in a ‘maternal’ figure, who enables the ‘unfolding of another life in relation to one’s own path towards death’, temporalities and practices of maintenance and waiting bound to repetition – a permanent labouring that goes on sustaining and ‘animat[ing] “life” in such a way as to allow the subject to die in its own fashion’ (Baraitser, 2020, 507, 503). Crucially, this repetition that sustains something unfolding in its own time and after its own fashion is a form of labour that is not a matter of indifference to the labouring subject; instead, ‘maternity’, writes Baraitser, ‘in its failure to be indifferent to the specificity of its labour, implies a return, again and again, to a scene that matters’ (509). Baraitser outlines here a time of repetition that keeps on coming back to what sustains life and the time it takes for lives to come to matter to one another – a time of waiting, of attention, and of care.

Rank’s account of the maternal seems, at first, to pull in another direction, as the death drive appears to turn away from the ongoing qualities of time: ‘the child envies the dead the happiness of return to the mother’ (Rank, 1929, 26), we hear. As the ultimate return to the maternal, death is a refuge from the trauma and anxiety of living through time: ‘At the moment of dying, the body once more severs itself from the mother substitute, “Dame World”’ (197). Rank even suggests that some of the phobic uncanniness associated with animals that creep into small holes is linked to the repressed desire to retreat into the maternal body, a ‘maternal hiding place’ (14). In ‘For to end yet again’, there is perhaps a cognate sense of retreat in the ‘refuge’ in which the figure is first encased. But, significantly, the refuge is not sustained as a state of quiescence; it comes to be called a ruin as it starts to disintegrate, and then a ‘mother ruin’ (Beckett, 1995, 245). Just as the return of the decrepit protagonist to his mother’s room in *Molloy* suggests a tomb that is also a womb from which the endless ending of the *Trilogy* is birthed, the ‘expelled’ is forced on from a seemingly still and inorganic state. The refuge/mother ruin in a desert mineral kingdom marks a return to the bedrock territory of the death drive as it lived in an asymptotic approach to the end. Although the aesthetic resources Beckett leaves himself are limited, there is always one more shudder of life, one more running through of this drive through matter, through form, in the only ever quasi ‘quiescence of the inorganic world’. ‘For to end yet again’ clearly represents a return to a scene that matters for Beckett – a ‘jerk back’ to make a ‘fresh start so as to prolong the journey’ (Freud, 1955a, 38) – in its repetitions of *Lessness* and a fundamentally lessened palette, exhausting itself through form, and iterating the ‘remains of the day’. Profoundly limited materials are arranged and rearranged, in different configurations of intensity – here as body, there as sand, there as rock. But the text also unfolds after its own fashion, carefully, over time, with minimal alterations to the scene in which shapes are shifted. While Beckett’s drama frequently stages the unavoidable return to relationality and waiting, even when all other accoutrements have been stripped away, in his late fiction the care of a ‘maternal’ death drive is figured as a sustained, prolonged return to a scene that matters (Salisbury, 2023a, 170), over and again, as particular configurations of materiality and of suffering, enduring corporeality emerge and recede, after their own fashion. Here, Beckett’s aesthetic care sustains the life of the work through iteration, by returning over and again, while also allowing the commitment to lessness and immanence in its pull away from progress, accumulation, gain, or transcendence.

In her recent work on what she names ‘geontology’, Elizabeth Povinelli articulates the fundamental biopolitical structure that has produced an extractive relationship to the non-human world within late liberalism: the distinction between Life and Nonlife, or we might say the organic and the inorganic, which becomes ‘the fundamental ground of the governance of difference and markets’ (Povinelli, 2016, 35). Here, the figure of the Desert becomes central, as Life drifts into the inorganic sands of Nonlife, awaiting actions that will reverse this movement, or preserve or protect it. But, aligning her work with ideas drawn from the Belyuen Indigenous community in Australia that complicate or refuse this as a fundamental organising principle, Povinelli describes a way of conceptualising ontology as the movements and relationships of change enabled at any given point or moment:

1. Things exist through an effort of mutual attention. This effort is not in the mind but in the activity of endurance.
2. Things are neither born nor die, though they can turn away from each other and change states.
3. In turning away from each other, entities withdraw care for each other. Thus the earth is not dying. But the earth may be turning away from certain forms of existence. In this way of thinking the Desert is not that in which life does not exist. A Desert is where a series of entities have withdrawn care for the kinds of entities humans are and thus has made humans into another form of existence: bone, mummy, ash, soil.
4. We must dedramatize human life as we squarely take responsibility for what we are doing. This simultaneous de-dramatization and responsibilization may allow for opening new questions. Rather than Life and Nonlife, we will ask what formations we are keeping in existence or extinguishing? (Povinelli, 2016, 28)

Povinelli’s geontology is not attempting to extend the ethical obligations that currently attend the Living to the geological, the inorganic, or the Nonliving; rather, it works to interfere with the organising principle of what currently underpins ethical and political understanding and action, to ‘de-dramatize’ the human as the source of action, intention, and sovereignty, and to think instead about the formations, or, we might say, the different configurations of intensity that enable a flourishing of life after its own fashion.^2^

‘For to end yet again’ might also be described as ‘de-dramatizing’ the human, with the shifting of its remains into ‘sand flowing’, and then out again, as the inorganic is bound into the organic (and vice versa) within this scene: ‘Grey cloudless sky grey sand as far as the eye can see long desert to begin […] Same grey all that little body from head to feet sunk ankle deep were it not for the eyes last bright of all […] Grey cloudless sky ocean of dust not a ripple’ (Beckett, 1995, 243–4). The flesh of the ‘Little body’ is becoming mineral, ‘marble still’, we hear repeatedly, just as much of the calcium carbonate that is in marble was once living – the shell and bone of sedimentary limestone – before it re-crystallised under heat and pressure. In Beckett’s text, life exists and persists, with continuing and even surprising shades of difference of intensity within the grey indifference. But in this waiting time there has been and continues to be a turning away from specific concentrations or forms of existence. The text dramatizes both a careful and repetitive turning back to a scene that matters and bodies that matter – suffering particularities – even as there is a steady and insistent withdrawal of care and attention from the kinds of entities that certain humans have imagined themselves to be, alongside the timelines that have sustained them.

## Unhappy Endings

There are no happy endings in Beckett’s work. There is no *kairotic* cut and then a tying up of ongoing time that would make an end that produces a sense of the fullness of time. But, in terms of the current times of catastrophic ecological losses, there is no invocation of an endurance that moves towards sufficiency and sustainability either. Caroline Levine has compellingly argued that the modernist tradition’s emphasis on the ‘open-ended pause’ ‘feels radical’, but ‘it delivers us from the responsibility to take action in the present’ (Levine, 2022, 389, 401). Against this, she writes in praise of novels that end by ‘focusing our attention on the mundane work of sustaining living bodies over time’ (391). By marking the end of a novel as a ‘threshold to sustainability’, we are invited to imagine lives going on beyond the end of the narrative world, tended by the repetitive ongoing work of care. This ‘narrative transition from precarity to sustainability’ (393) that Levine finds invoked at the end of some nineteenth-century realist novels, this suggestion of the happy ending that asserts the potential and need for making a good and sustaining life in the here and now, could hardly be further from Beckett’s aesthetic, even as much of his drama emphasises the ongoing time and care of selves that remain in contact with others, waiting with and on one another. Beckett’s own invocation of ‘sustained and sustaining routines’ does not enable flourishing in the way Levine suggests (396), but instead dramatizes increasingly minimised and lessened ways of going on, with the work of the death drive enduring as the bedrock to which all is turning and returning. But we might note that Levine’s turn to domestic sustenance and the pleasures imagined in nineteenth and twentieth-century novels appears to avert its eyes, at least partially, from the expansion, increased production, and the accelerations of that period – the extractive exploitation of new territories and development of new markets in which sustenance for some meant a withdrawal of care for the conditions of others. What Beckett’s mid-to-late work offers through the time of the ‘Great Acceleration’ is a withdrawal of care and attention from those modes of accumulation and gain, a willed turn to ‘lessness’, and an insistence instead on paying attention to the depletion and the suffering finitude of organisms seeking to return to ‘inorganic existence’ in ways ‘immanent in the organism itself’ (Freud, 1955a, 38). Care and attention are offered to the inorganic, too, as it shifts, changing state after its own particular fashion.

This is neither a happy ending nor a way of preserving the world that articulates what is to be done drawn from a *kairotic* end. No: if an ethics or even a politics for the ‘end times’ is to be drawn from Beckett’s late vision, it merely insists on showing the binding of non-life within life, the inorganic within the organic, the mineral within flesh and vice versa, that de-dramatizes and decentres a specific formation of the human. Staging the inevitable turning away and withdrawal of care for the human when glimpsed across vast tracts of geological time, the future is viewed not as an open horizon for ‘us’; instead, the late texts pay attention to those intensive temporalities that go on alongside but also without us. Meanwhile, something remains: specificities of suffering corporeality and lives and timelines that need witnessing and tending – that need attending to – endure in the meantime. There is nothing to do but wait; but what happens while waiting is the repetition of attention and care in the return to a scene that matters. Such repetition sustains something for a time in order that it might die after its own fashion, rather than be killed or wilfully allowed to perish before its time – that fate persistently suffered by human populations who are hardly registered as living at all, or the more-than-human world that might simply look to exist in its own time rather than be destroyed. To wait with Beckett is to pay attention to the undertow of the timelines of gain and progress that have marked the globe and hold back from the ‘trop vite’ that risks mortgaging the existence of many human and more-than-human others to the sustaining of a certain configuration of life only. Beckett’s vision won’t save ‘us’; after all, when you’re ‘on earth, there’s no cure for that’ (Beckett, 1990, 125). But something remains nevertheless – a glimpse of the care that can persist in the meanwhile.

## Notes

1 For a reading of grey as the temporality of ‘afterness’, see Tsushima (2023).

2 In his account of ‘animist time’ as something that interrupts and decentres the White Anthropocene, Allen-Paisant draws on and cites Aimé Césaire’s respect for the vegetal, mineral and animal within us as a manifestation of a sense of ‘animist time’, one which offers a way out of reification and ‘a world of efficiency, in which man himself becomes a thing, a world where time is no longer time, but a kind of space, filled with qualitatively measurable things’ (Allen-Paisant, 2021, 34).
